# Impact of January 2021 curfew measures on SARS-CoV-2 B.1.1.7 circulation in France

**DOI:** 10.2807/1560-7917.ES.2021.26.15.2100272

**Published:** 2021-04-15

**Authors:** Laura Di Domenico, Chiara E Sabbatini, Giulia Pullano, Daniel Lévy-Bruhl, Vittoria Colizza

**Affiliations:** 1 INSERM, Sorbonne Université, Pierre Louis Institute of Epidemiology and Public Health, Paris, France; 2 Orange Labs, Sociology and Economics of Networks and Services (SENSE), Chatillon, France; 3 Santé publique France, Saint-Maurice, France; 4 Tokyo Tech World Research Hub Initiative, Institute of Innovative Research, Tokyo Institute of Technology, Tokyo, Japan

**Keywords:** SARS-CoV-2, B.1.1.7, variants, modelling, social distancing, curfew

## Abstract

Following the spread of the SARS-CoV-2 B.1.1.7 variant, social distancing was strengthened in France in January 2021. Using a two-strain mathematical model calibrated on genomic surveillance, we estimated that curfew measures allowed hospitalisations to plateau by decreasing transmission of the historical strains while B.1.1.7 continued to grow. School holidays appear to have further slowed down progression in February. Without progressively strengthened social distancing, a rapid surge of hospitalisations is expected, despite the foreseen increase in vaccination rhythm.

The new B.1.1.7 variant of severe acute respiratory syndrome coronavirus 2 (SARS-CoV-2) (20I/501Y.V1, also called variant of concern (VOC) 202012/01) initially detected in the United Kingdom [1,2] has rapidly expanded its geographical range across European countries [3]. A large-scale genome sequencing initiative was conducted in France on 7–8 January (Flash1 survey [4], the first of a set of surveys), reporting that 3.3% of all SARS-CoV-2 detections were B.1.1.7 viruses. To limit SARS-CoV-2 spread, strengthened social distancing measures were implemented in the country in the month of January. Starting from a curfew at 20:00 in place since mid-December, the national authorities set a curfew at 18:00 from 2 January in several departments with deteriorating indicators. This was extended nationwide on 16 January, with renewed recommendations on teleworking and preventive measures. On 31 January, stricter controls of the compliance with the measures and closure of large commercial centres were applied.

The presence of the B.1.1.7 variant on the territory, however, poses critical challenges to epidemic control. Its higher transmissibility represents a strong selective advantage that makes it prone to rapidly becoming the dominant strain [1,2,4-8]. Social distancing has a differential impact on the variant and the historical strains, not visible before the implementation of surveillance that monitored variant frequency over time. Assessing the impact of implemented measures on the two strains through modelling is key for epidemic management.

## Modelling SARS-CoV-2 two-strain transmission dynamics

We extended a previously developed age-stratified transmission model that was used to assess the impact of interventions against the coronavirus disease (COVID-19) pandemic in France in 2020 [9-11], fitted to hospital admission data and validated against the estimates from serological studies [9]. The model is discrete, stochastic, and integrates demography, age profile, social contacts and mobility data over time to account for social distancing measures. Details are provided in [9] and in the Supplement.

The model was extended to describe the circulation of two SARS-CoV-2 variants – the historical strains and B.1.1.7. Variant circulation was initialised on Flash1 data [4]: France (3.3%), the Île-de-France region reporting the highest penetration (6.9%) and the Nouvelle Aquitaine region reporting one of the lowest penetrations (1.7%). We considered a 59% higher transmissibility (95% confidence interval (CI): 54–65) for B.1.1.7 estimated for France on Flash1 and Flash2 survey data [4] in line with previous estimates [1,2] and assumed complete cross-immunity [1,2].

The model was fitted to daily hospital admission data in each territory to evaluate the impact of curfew in January (weeks 2–5) and of curfew and school holidays in February (weeks 6–9, with regional calendars: weeks 6–7 in Nouvelle Aquitaine, weeks 7–8 in Île-de-France). We projected future trends in hospitalisations at the end of the holidays, assuming the estimated curfew conditions. We also considered two scenarios corresponding to the strengthening and relaxation of social distancing measures, obtained with, respectively, a 15% reduction and increase of the effective reproductive number estimated for the curfew.

Vaccination prioritised to older age groups was simulated according to the daily rhythm of 100,000 vaccine doses administered per day as recorded in February [12] and then increased to 200,000 (first) doses per day (accelerated rhythm) from week 10 following government announcements (Supplement) [13]. This vaccination roll-out was compared for sensitivity with an optimistic rhythm of 300,000 (first) doses per day from week 10, and with a stable rhythm maintaining the administration of 100,000 doses per day over time.

## Estimated impact of social distancing measures and resulting B.1.1.7 trends

**Figure 1:**
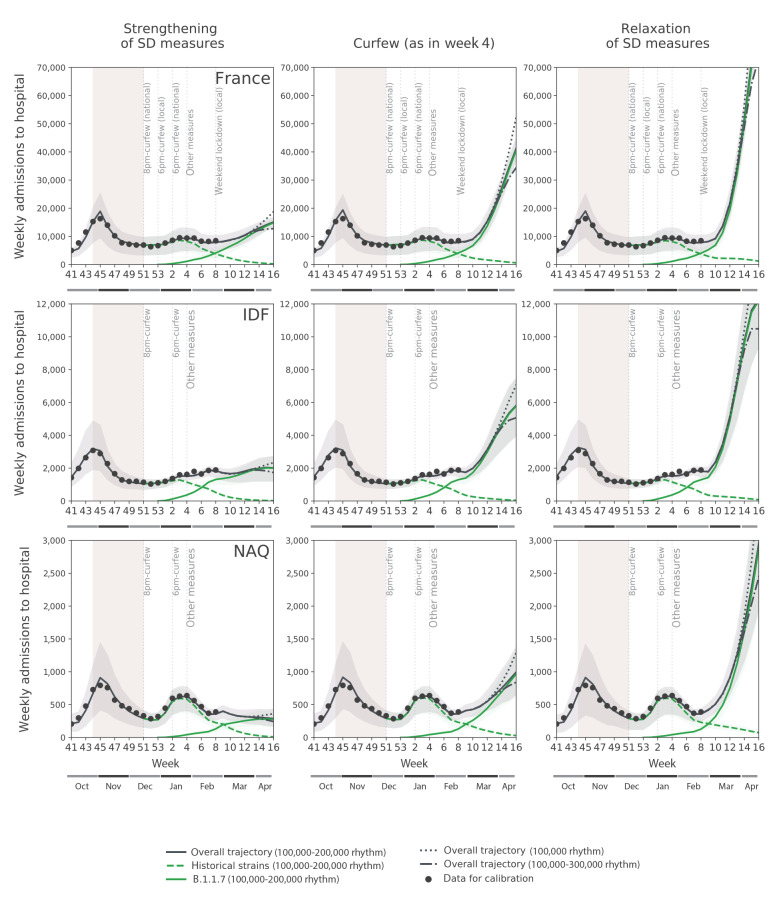
Projected weekly hospital admissions due to SARS-CoV-2 historical strains and B.1.1.7 variant in France and two French regions, October 2020–April 2021

IDF: Île-de-France; NAQ: Nouvelle Aquitaine; SARS-CoV-2: severe acute respiratory syndrome coronavirus 2; SD: social distancing.

After an increase in registered hospital admissions from December (average 6,700 weekly hospitalisations at national level) to early January (ca 9,000 in week 2), the epidemic plateaued in the second half of January, following increased restrictions. Based on the estimated prevalence of the B.1.1.7 variant on 7–8 January yielded by the Flash survey and on the reported hospitalisations in weeks 2–5, the model explains this plateau as the counterbalance between two opposing dynamics: a decreasing circulation of the historical strains (with effective reproductive numbers *R_e_^FR^* = 0.96 (95% CI: 0.95–0.97), *R_e_^IDF^* = 0.90 (95% CI: 0.86–0.93) and *R_e_^NAQ^* = 0.84 (95% CI: 0.77–0.90) in week 4 for France (FR), Île-de-France (IDF) and Nouvelle Aquitaine (NAQ), respectively) vs the exponential increase of the variant (Figure 1). Curfew and other social distancing measures reduced the reproductive number of the historical strains below 1, but they were not enough to prevent the increasing B.1.1.7 dynamics. The estimated *R_e_* for the B.1.1.7 variant was largely above 1 in all regions: *R_e_^FR^* = 1.53 (95% CI: 1.51–1.54), *R_e_^IDF^* = 1.43 (95% CI: 1.37–1.48), *R_e_^NAQ^* = 1.34 (95% CI: 1.22–1.43). School holidays further slowed down the historical strains, with *R_e_^FR^* = 0.78 (95% CI: 0.77–0.79) and *R_e_^IDF^* = 0.64 (95% CI: 0.62–0.67) in week 8 and *R_e_^NAQ^* = 0.65 (95% CI: 0.62–0.68) in week 7, but their effect was still insufficient against the variant (median *R_e_^FR^* > 1 for the B.1.1.7 variant in all territories).

From left to right, different scenarios after the winter school holidays are considered: strengthening of SD measures scenario, equivalent to the second lockdown; curfew scenario, estimated in week 4 and assuming no additional changes; relaxation of SD measures scenario, compatible with the situation at the start of the year before increased restrictions.

From top to bottom: the different regions considered.

The solid grey curve refers to the median overall trajectory, obtained under the accelerated vaccination roll-out (100,000–200,000 doses/day) and because of the concurrent circulation of the historical strains (dashed green curve) and the B.1.1.7 variant (solid green curve) assumed to have a 59% higher transmissibility (median value is estimated for France; increases corresponding to the 95% confidence intervals of this estimate are reported in the Supplement). A slower (100,000, dotted curve) and an optimistic (100,000–300,000, dot-dashed curve) vaccination rhythm are also shown (only median curves of the overall trajectories are shown, for the sake of visualisation). The shaded area around the curves corresponds to the 95% probability range obtained from 500 stochastic simulations. Dots correspond to weekly hospital admission data. The model is fitted to daily hospital admissions since the start of the epidemic, propagating uncertainty over time; the figure shows weekly data to simplify the visualisation. The second wave is shown for reference, together with indications of the timing of social distancing measures; the shaded rectangle around the second wave corresponds to the second lockdown. Months are indicated at the bottom of the x-axis (from October 2020 to April 2021). The results do not integrate the effect of more stringent measures recently implemented to curb the third wave, and do not include Easter school holidays, nor seasonal effects.

**Figure 2:**
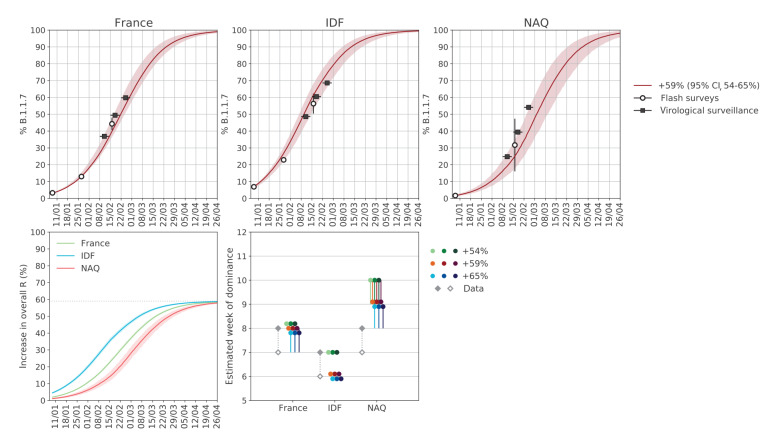
Projected prevalence of SARS-CoV-2 variant B.1.1.7 over time and estimated week when B.1.1.7 becomes the dominant strain in France and two French regions, 11 January–26 April 2021

CI: confidence interval; IDF: Île-de-France; NAQ: Nouvelle Aquitaine; R: reproductive number; SARS-CoV-2: severe acute respiratory syndrome coronavirus 2.

The projected increase in B.1.1.7 prevalence over time was confirmed by sequence data in the Flash2 and Flash3 surveys (conducted on 27 January and 16 February, respectively [4,14]) and by weekly virological surveillance data available starting week 6 detecting mutations specific to the variants of concern (Figure 2; Supplement). The data also matched the estimated date of B.1.1.7 dominance, showing that B.1.1.7 accounted for the majority of cases by week 8 in France and Nouvelle Aquitaine and by week 7 in Île-de-France. The variant was expected to increase by more than 55% the overall effective reproductive number by 18 March in Île-de-France, by 30 March in France and by 4 April in Nouvelle Aquitaine, compared with a situation without the variant.

Top: estimated percentage of B.1.1.7 cases over time, considering a 59% (95% CI: 54–65) higher transmissibility for the variant. From left to right: the different regions considered.

Circles represent the estimates from the genomic surveillance in the Flash surveys (Flash1 on 7–8 January, Flash2 on 27 January, Flash3 on 16 February). The Flash2 estimate was not available for Nouvelle Aquitaine, as sequencing failed or was not performed on the majority of samples, preventing a reliable estimate. Squares represent results from weekly virological surveillance screening allowing the detection of the N501Y mutation specific to the B.1.1.7 variant (Supplement). We estimated 95% CI assuming a normal distribution. Flash3 survey estimates have larger CI as sequencing was performed on a smaller sample of viruses. Horizontal bars in weekly virological surveillance correspond to the week of reference.

Bottom left: percentage increase in the overall effective reproductive number at the population level following an increased penetration of the variant, assuming a 59% higher transmissibility of the variant. Curves represent median values; shaded areas around the curves represent 95% probability ranges obtained from 500 stochastic simulations.

Bottom right: estimated week of B.1.1.7 dominance, assuming a 59% (95% CI: 54–65) higher transmissibility for the variant, and considering the curfew scenario (middle point) and the scenarios with strengthening (lighter colour, left point) and relaxation (darker colour, right) of social distancing measures. Error bars represent 95% probability ranges. Grey diamonds correspond to the last week when the reported frequency was < 50% (open symbol) and to the first week with reported frequency > 50% (filled symbol).

## Projected hospitalisations under different scenarios

Assuming that the epidemic progressed under the estimated epidemiological conditions of the curfew, and if vaccination was accelerated as announced, the model predicted that hospitalisation levels similar to the peak in November 2020 (close to hospital capacity in a number of regions) would be reached around week 13 in France, week 12 in Île-de-France and week 15 in Nouvelle Aquitaine (Table). This was later confirmed by data, reporting that COVID-19 hospitalisations exceeded the peak of the second wave in week 12 in Île-de-France [15], then triggering more stringent interventions to curb the third wave. Under a partial relaxation of social distancing – approximately corresponding to the situation at the turn of the year before stricter measures were implemented in January 2021 – these hospitalisation levels were expected to be reached at least 1 week sooner. Implementation of stronger social distancing immediately after school holidays, equivalent to the second lockdown, were predicted to maintain hospitalisations below the peak of the second wave in Île-de-France and Nouvelle Aquitaine when assuming the median estimate for the transmissibility advantage of the variant. However, this scenario predicted that a rise of hospitalisations comparable to the second wave was possible in France even under the accelerated vaccination rhythm (100,000–200,000 doses/day). Accelerated and optimistic vaccination roll-out would reduce weekly hospitalisations by, respectively, ca 20% and 35% in week 16 compared with a stable vaccination campaign without acceleration.

**Table:** Estimated week when COVID-19 hospitalisations will exceed the peak of the second wave in France and two French regions, March–May 2021

Peak weekly hospitalisations in the second wave		B.1.1.7 transmissibility advantage	Strengthening of SD measures	Curfew (as in week 4 of 2021)	Relaxation of SD measures
France	16,000 weekly hospitalisations	54%	NR	Week 13 (12–14)	Week 12 (11–12)
		59%	After week 15	Week 13 (12–13)	Week 12 (11–12)
		65%	Week 15 (13–16)	Week 12 (11–13)	Week 12 (11–12)
Île-de-France	3,000 weekly hospitalisations	54%	NR	Week 12 (11–12)	Week 11 (11–12)
		59%	NR	Week 12 (11–12)	Week 11 (11–12)
		65%	NR	Week 11 (11–12)	Week 11 (11–12)
Nouvelle Aquitaine	800 weekly hospitalisations	54%	NR	Week 16 (14–20)	Week 12 (12–14)
		59%	NR	Week 15 (13–19)	Week 12 (11–13)
		65%	Week 15 (12–17)	Week 13 (12–15)	Week 11 (11–12)

COVID-19: coronavirus disease; NR: not reached; SD: social distancing.

Projections after winter school holidays consider three scenarios. From left to right: strengthening of SD measures scenario, equivalent to the second lockdown in November 2020; curfew scenario, estimated in week 4 and assuming no additional changes; relaxation of SD measures scenario, compatible with the situation at the start of 2021 before increased restrictions. Results correspond to a 59%, 54% and 65% higher transmissibility of the variant compared with the previously circulating virus (median and values of the 95% confidence interval of the estimate for France) and to the 100,000–200,000 daily doses rhythm (see Supplement for details and results from other daily rhythms). Uncertainties (in brackets) refer to 95% probability ranges; NR indicates that the peak level is not predicted to be reached before week 16, the end of the time period under study. The results do not integrate the effect of more stringent measures recently implemented to curb the third wave, and do not include Easter school holidays, nor seasonal effects.

## Discussion

We estimated that social distancing progressively implemented at the start of January 2021 was able to bring the effective reproductive number of the historical SARS-CoV-2 strains below 1, leading to its decline, while B.1.1.7 cases increased exponentially. School holidays in February slowed down the dynamics further. The predicted growth in this variant’s frequency and the date when it became the dominant strain matched recent data.

Social distancing was the combined effect of imposed restrictions [16] and individual responses to renewed recommendations on teleworking and risk reduction. Teleworking, estimated from mobility data [9,17], was maintained in January at the levels reached before releasing the second lockdown. Measures, however, were not enough to lead to a decline in the variant spread, not even under the additional impact of holidays, owing to this variant’s more efficient transmission.

Strengthening social distancing through a mild lockdown, such as the one implemented to curb the second wave in November 2020, was predicted to allow certain regions to avoid a third wave of the same magnitude of the second, supported by acquired immunity (Île-de-France) or lower incidence levels (Nouvelle Aquitaine, having achieved a marked decrease in hospitalisations in February). The lockdown in November 2020 included restrictions on mobility, closure of non-essential shops, while school at all levels remained open. In our model, however, the strengthening of social distancing measures was optimistically implemented immediately after school holidays in February and with a duration longer than a month. In the absence of these early measures, the model predicted that curfew alone would not be sufficient to prevent a rapid resurgence of hospitalisations, as was later confirmed by the rising third wave in France in March 2021. Projections on the week exceeding hospitalisation levels of the second peak in Île-de-France matched observations [15] before the authorities applied more stringent measures in the region and other territories on 20 March and extended them nationwide at the end of the month. 

Our study has limitations. Results are based on the estimated impact of curfew and scenarios anticipating a possible strengthening or relaxation of social distancing. We did not consider changes in behaviour such as a progressive abandoning of teleworking because of fatigue or increased risk prevention triggered by growing concern. We could not yet include the impact of more stringent measures recently put in place to curb the third wave that will inevitably alter the projected dynamics from the end of March. This will also affect projections of the national model, unable to account for geographically targeted interventions put in place on 20 March. Our analysis based on the estimated transmissibility advantage of B.1.1.7 at the national level [4] identified differences between the two regions Île-de-France and Nouvelle Aquitaine. These could be partly due to biases affecting Flash survey data and linked to reinforced tracing around suspected or confirmed variants. These biases are expected to be stronger in regions with small epidemics (Nouvelle Aquitaine) than in regions with higher incidence levels and variant penetration (Île-de-France). Also, small sample sizes in Nouvelle Aquitaine increase uncertainty around the estimates. We did not consider in the main analysis additional differences between the variant and the historical strains beyond the transmissibility advantage. The recently estimated increased hospitalisation rate associated with B.1.1.7 infection [18] would lead to a higher peak in projected hospitalisations at the end of April 2021, after the period under study here (Supplement). We did not consider other variants that were estimated to have a lower penetration, but their circulation is likely to contribute to the expected surge in cases [19]. 

Accelerating vaccination roll-out is key [20], but even optimistic roll-out plans would require more rigorous and intensified social distancing than curfew alone to curb the B.1.1.7 epidemic. 

## Supplementary Data

8203000 Supplement
