# Supplementary material for: Impact of January 2021 curfew measures on SARS-CoV-2 B.1.1.7 circulation in France
Source: Euro Surveill. 2021 Apr 15;26(15):2100272. doi: 10.2807/1560-7917.ES.2021.26.15.2100272 (PMC8167415; doi:10.2807/1560-7917.ES.2021.26.15.2100272)
Supplement: Supplement [file 21-00272_COLIZZA_Supplement.pdf]

Supplementary Material

Impact of January 2021 curfew measures on SARS-CoV-2 B.1.1.7 circulation in France

Laura Di Domenico[1], Chiara E. Sabbatini[1], Giulia Pullano[1,2], Daniel Lévy-Bruhl[3], Vittoria Colizza[1,4,*]

[1]*INSERM, Sorbonne Université, Pierre Louis Institute of Epidemiology and Public Health, Paris, France*
[2]*Orange Labs, Sociology and Economics of Networks and Services (SENSE), Chatillon, France*
[3]*Santé publique France, Saint-Maurice, France*
[4]*Tokyo Tech World Research Hub Initiative, Institute of Innovative Research, Tokyo Institute of Technology, Tokyo, Japan*

This text provides: a description of the data used in the study; details on the transmission model and inference framework; additional results and sensitivity analysis.

This supplementary material is hosted by Eurosurveillance as supporting information alongside the article "*Impact of January 2021 curfew measures on SARS-CoV-2 B.1.1.7 circulation in France*", on behalf of the authors, who remain responsible for the accuracy and appropriateness of the content. The same standards for ethics, copyright, attributions and permissions as for the article apply. Supplements are not edited by Eurosurveillance and the journal is not responsible for the maintenance of any links or email addresses provided therein.

1. Surveillance data 2
1.1. Hospital surveillance data 2
1.2. Virological and genomic surveillance data 2
2. SARS-CoV-2 two-strain transmission model 3
2.1. Compartmental model and parameters 3
2.2. Generation time distribution 4
2.3. Parameterization of contact matrices from empirical data 5
2.4. B.1.1.7 variant 6
2.5. Vaccination rollout campaign 7
3. Inference framework 7
4. Additional results and sensitivity analysis 8
4.1. Impact of B.1.1.7 transmissibility advantage 8
4.2. Impact of strengthening / relaxation of social distancing measures on projected B.1.1.7 prevalence 10
4.3. Impact of more restrictive social distancing measures on projected trends of hospital admissions 10
4.4. Sensitivity analysis on vaccine daily rhythm and efficacy 11
4.4.1. Impact of vaccination rhythms on the projected weekly hospitalizations 11
4.4.2. Impact of vaccine efficacy on the projected weekly hospitalizations 13
4.5. Impact of increased hospitalization rates associated to B.1.1.7 infection 13
5. References 14

1. Surveillance data

1.1. Hospital surveillance data

Our model was fitted to daily hospital admission data to capture the epidemic trajectory over time (see the Inference section). We use the SIVIC database which lists daily hospitalizations of patients for COVID-19 (cases confirmed by PCR or chest CT) in public or private hospitals in France. They are among the most robust data sources to use in COVID-19 epidemiological studies, as they are not affected by changes in detection and sampling, as it happens for detected cases, and suffer less the delays or uncertainty in classification of the number of deaths. These data have been used throughout 2020 in France to respond to the health crisis[1–3] and are also routinely used by health agencies for their assessments of the epidemiological situations.

Data include admissions to conventional hospitalizations or critical care for COVID-19 by date of event (admission, entry into critical care) and not by date of registration of the event in the database, and are corrected for notification delays. They exclude transfer of patients, follow-ups, rehabilitation care. As such, data may be different from those reported in official statistics.

1.2. Virological and genomic surveillance data

Genome sequencing to estimate the frequency of variants of concern at different moments in time was conducted in France through three surveys, called Flash surveys. They adopted different protocols, evolving over time due to the urgency of assessing the circulation of the variants and because of logistical and resource constraints.

Flash1 and Flash2 surveys were large-scale genome sequencing initiatives, analyzing all samples provided by participating laboratories at given dates. Flash 1 survey[4] analyzed through Thermo Fisher 11,916 PCR positive samples out of 183,363 samples collected on January 7-8. 298 samples were confirmed B.1.1.7 variant through sequencing, corresponding to 70% of analyzed S-gene dropouts, leading to 3.3% of new cases on January 7-8 due to SARS-CoV-2 B.1.1.7 variant in France. The estimated proportion of B.1.1.7 in Île-de-France and Nouvelle Aquitaine was 6.9% and 1.7%, respectively[4]. Flash2 survey analyzed 10,261 PCR positive samples out of 120,335 samples collected on January 27 by participating laboratories[4]. 3,561 positive samples were analyzed through Thermo Fisher using the same protocol of Flash1, and S-gene target failure was identified in 475 PCR positive samples. 261 samples were confirmed B.1.1.7 variant, corresponding to 83.6% of S-gene dropouts analyzed through sequencing. The remaining PCR positive samples were analyzed through an alternative screening protocol and then sequenced, resulting in 946 confirmed B.1.1.7 viruses, corresponding to a PPV equal to 100%[4]. The alternative screening protocol was based on second-line RT-PCR tests with specific primers that allow the detection of the main mutations that characterize the variants of concern. They must include at least the N501Y mutation and allow to distinguish the 20I/501Y.V1 variant from the 20H/501Y.V2 or 20J/501Y.V3 variants. The list of second-line RT-PCR kits authorized by the French Drug Agency (ANSM) is available on the Ministry of Health website[5]. This protocol was in place since January 25. Estimated B.1.1.7 penetration in Flash2 survey was 13.0% in France and 22.9% in Ile-de-France[5]. In Nouvelle Aquitaine, data for more than 50% of positive samples resulting from the two types of screening could not be used because sequencing failed or was not performed, preventing a reliable estimate of B.1.1.7 frequency given the limited number of screened samples (50). Flash3 survey was conducted in week 6 and was based on a smaller sample of sequences to reduce time and resources needed for sequencing: 699 in France, 267 in Ile-de-France, 34 in Nouvelle Aquitaine[6]. After imputing sequencing that could not be interpreted (12%, 13%, 18%, respectively), B.1.1.7 frequency was estimated to be: 44.3% in France, 56.3% in Ile-de-France, 31.7% in Nouvelle Aquitaine. Values from Flash1, Flash2, Flash3 surveys are reported in Figure 2 of the main paper. Their confidence interval is estimated assuming that B.1.1.7 frequency is described by a normal distribution.

Given the heavy load of sequencing, and the need to provide more timely indicators on the circulation of the variants of concern, starting week 6 a new protocol for virological surveillance was implemented to provide estimates on the weekly frequency of detected viruses with specific mutations[7], using the second-line RT-PCR tests described above. In Table S1 and Figure 2 of the main paper we report the proportion of positive screened samples showing the N501Y mutation specific to B.1.1.7 variant. PPV value was 100% with this type of screening during Flash3 survey, conducted during week 6, making this a reliable proxy to monitor B.1.1.7 frequency over time.

Table S1. Proportion of samples showing the N501Y mutation specific to B.1.1.7 variant. Data accessed on March 5.

	France	IDF	NAQ
Week 6	36.9%	48.6%	24.8%
Week 7	49.4%	60.5%	39.4%
Week 8	59.8%	68.6%	54.1%

2. SARS-CoV-2 two-strain transmission model

2.1. Compartmental model and parameters

We use a stochastic discrete age-stratified transmission model, integrating demographic, age profile, social contact data, mobility data, data on adoption of preventive measures, to account for age-specific behaviors over time and role in COVID-19 transmission. Four age classes are considered: [0-11), [11-19), [19-65), and 65+ years old (children, adolescents, adults, seniors). Transmission dynamics follows a compartmental scheme specific for COVID-19 (**Figure S1**) where individuals are divided into susceptible, exposed, infectious, hospitalized and recovered. The infectious phase is divided into two steps: a prodromic phase (I_p) and a phase where individuals may remain either asymptomatic (I_{as}, with probability p_a=40%[8]) or develop symptoms. In the latter case, we distinguished between different degrees of severity of symptoms (paucisymptomatic (I_{ps}), individuals with mild symptoms (I_{ms}), or severe symptoms (I_{ss}) requiring hospitalization[3,9,10]). Prodromic, asymptomatic and paucisymptomatic individuals have a reduced transmissibility[11]. A reduced susceptibility was considered for children and adolescents, along with a reduced relative transmissibility of children based on available evidence[12–17]. We assume that infectious individuals with severe symptoms reduce of 75% their number of contacts because of the illness they experience[18]. Parameter values are reported in **Table S2**.

Sensitivity analysis on the probability of becoming symptomatic and the transmissibility of children was performed in previous work[1,2,19].

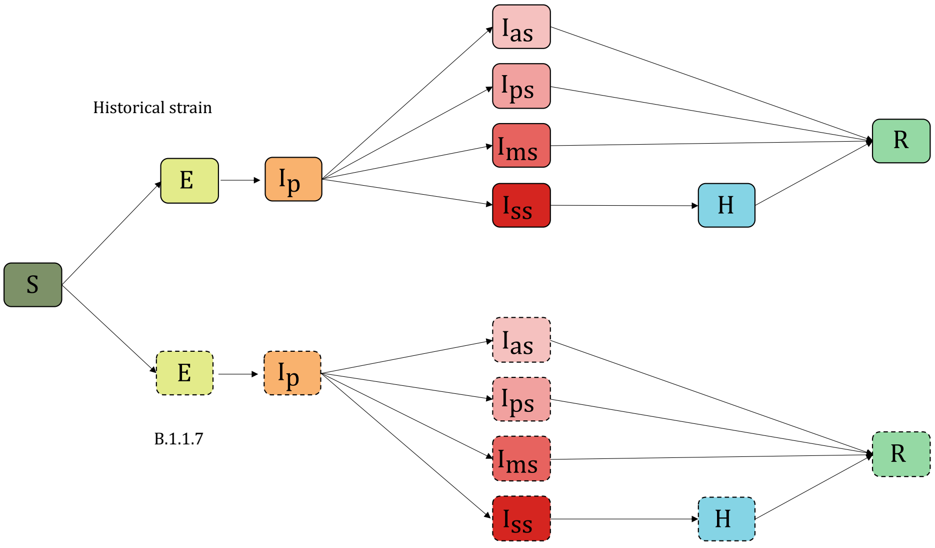

Figure S1. Two-strains compartmental scheme. Compartments with continuous line (top) account for the diffusion of historical strain, compartments with dashed line (bottom) account for the diffusion of B.1.1.7 variant. S=Susceptible, E=Exposed, I_p= Infectious in the prodromic phase, I_{as}=Asymptomatic Infectious, I_{ps}=Paucysymptomatic Infectious, I_{ms}=Symptomatic Infectious with mild symptoms, I_{ss}=Symptomatic Infectious with severe symptoms, H=severe case admitted to the hospital, R=recovered.

Table S2. Parameters, values, and sources used to define the compartmental model.

Variable	Description	Value	Source
θ^{-1}	Incubation period	5.2d	[20]
μ_p^{-1}	Duration of prodromal phase	1.5d, computed as the fraction of pre-symptomatic transmission events out of pre-symptomatic plus symptomatic transmission events.	[21]
ϵ^{-1}	Latency period	$\theta^{-1} - \mu_p^{-1}$	-
p_a	Probability of being asymptomatic	0.4	[8]
p_{ps}	If symptomatic, probability of being paucisymptomatic	1 for children, adolescents<br>0.2 for adults, seniors	[9]
p_{ms}	If symptomatic, probability of developing mild symptoms	0 for children, adolescents<br>0.7704 for adults<br>0.546 for seniors	[3,9,10]
p_{ss}	If symptomatic, probability of developing severe symptoms	0 for children, adolescents<br>0.0296 for adults<br>0.254 for seniors	[3,10]
g	Generation time	6.6d	[22]
μ^{-1}	Infectious period	2.3d, chosen accordingly to generation time distribution	-
r_β	Relative infectiousness of I_p, I_a, I_{ps}	0.25 for children<br>0.55 for adolescents, adults, seniors	[11]
s	Relative susceptibility	0.5 for children, adolescents<br>1 for adults, seniors	[13]

2.2. Generation time distribution

The generation time distribution was computed based on the approach of Ref.[23]. Let X and Y be the random variables describing the latency period and the infectious period, respectively. Then the distribution of the generation time is the result of the convolution $g * h_s$, with g being the probability density function of X and

$$h_s(t) = \frac{1 - H(t)}{E(Y)}$$

where H is the cumulative distribution function of Y, and $E(Y)$ is the mean.

In the compartmental model under consideration (**Figure S1**), we have that X is exponentially distributed with rate ϵ, and Y is the sum of two exponentially distributed random variables (prodromic phase and infectious period, with rate μ_p and μ respectively). Computations show that the corresponding generation time distribution is

$$f(t) = \frac{\epsilon\, \mu_p\, \mu}{(\mu_p + \mu)(\mu - \mu_p)} \left[\frac{\mu}{(\epsilon - \mu_p)} \left(e^{-\mu_p t} - e^{-\epsilon\, t}\right) - \frac{\mu_p}{(\epsilon - \mu)} \left(e^{-\mu\, t} - e^{-\epsilon\, t}\right)\right]$$

Given the values of ϵ and μ_p informed from the literature (**Table S2**), we chose μ so that the mean of the generation time equals to 6.6 days. The shape of the distribution is displayed in **Figure S2** and it closely resembles a gamma distribution with mean 6.6 and shape parameter 1.87, estimated in Ref[22].

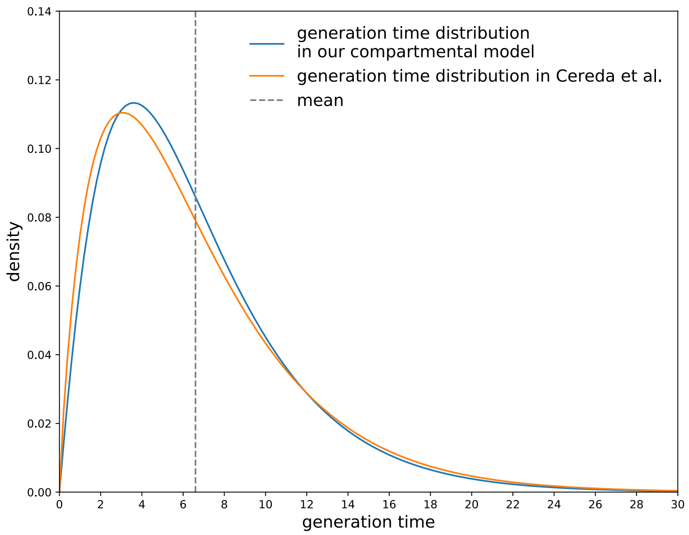

Figure S2. Distribution of the generation time. The generation time distribution corresponding to our compartmental model (blue) in comparison with the distribution estimated in Ref.[22] (orange).

2.3. Parameterization of contact matrices from empirical data

Social mixing was informed from behavioral data and was modeled through the parametrization of contact matrices. In particular we considered attendance at school[24], percentage of telework[25], and adoption of physical distancing over time[26].

Contacts at school were considered according to the school calendar. In France all schools are in session with 100% physical presence since the start of the school calendar in September. In the period of May-July, after the first lockdown, schools were open but attendance was on a voluntary basis[19].

Social contacts at work were modified to account for the percentage of workers not going to their place of work over time, following the variation of presence at workplaces based on Google Mobility Trends[25] (**Figure S3**).

To account for individuals' risk protection behavior over time, we parametrized contact matrices with the percentage of population avoiding physical contacts from the results of regular large-scale surveys conducted by Santé Publique France (CoviPrev[26]). From these data, we also estimated that seniors have a higher risk aversion behavior compared to other age classes, leading to an average additional 30% reduction of their physical contacts[1].

The contacts in leisure and non-essential activities were informed based on implemented restrictions and mobility data in community settings (see e.g. use of transport and visits to retail in **Figure S4**). A sensitivity analyses on contacts in leisure and non-essential activities was conducted in Ref.[1].

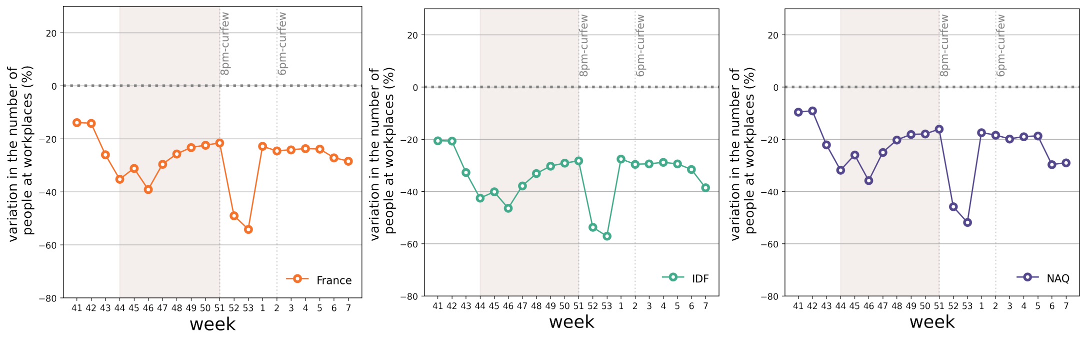

Figure S3. Estimated change in presence at workplace locations over time and by region based on Google location history data[25].

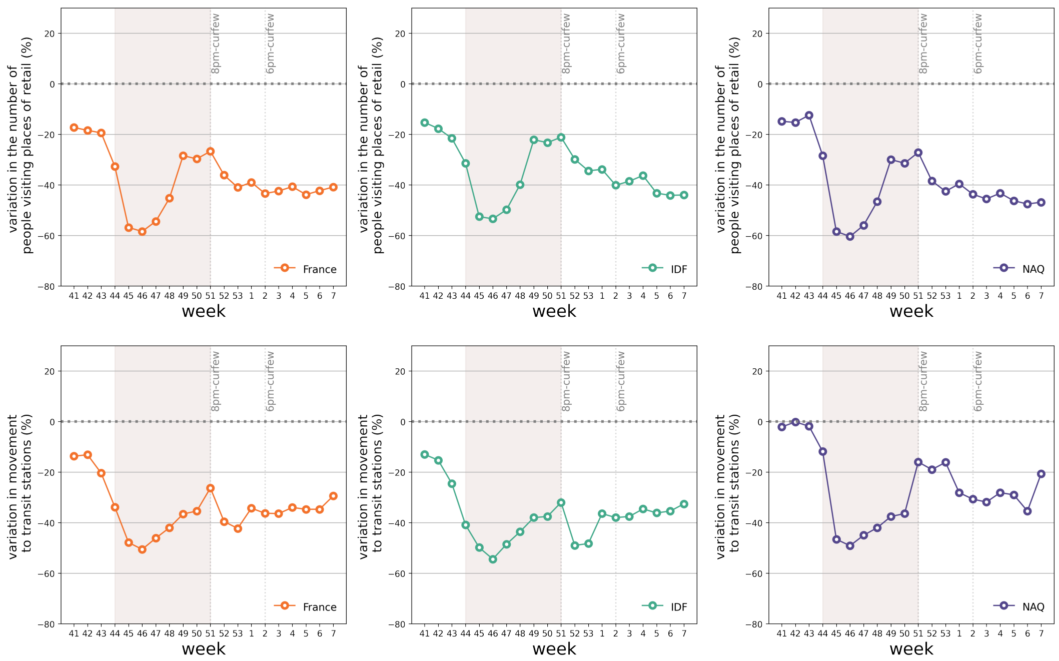

Figure S4. Estimated change in the number of people visiting places of retail (top) and in the movement to transit stations (bottom) over time and by region based on Google location history data[25].

In prior work[1], we compared our approach where the contact matrix is parameterized with the various data sources described above with a simplified version of the model that neglects these input data. This version assumes that all changes in the epidemic trajectory are absorbed exclusively by the transmissibility per contact. This is equivalent to normalize the contact matrix to its largest eigenvalue and estimate the reproductive ratio over time, as also done in other works[3]. Results showed that our model better describes the observed trajectories, thus indicating that changes in age-stratified contact patterns are important to capture the epidemic dynamics.

The code for the model is available at the link provided in Ref.[1].

2.4. B.1.1.7 variant

We considered the co-circulation of B.1.1.7 variant together with the historical strain. Complete cross-immunity and 59% (95% CI: 54-65%)[4] increased transmissibility were considered for B.1.1.7 variant compared to the historical strain (**Figure S1)**. This estimate was obtained from the Flash1 and Flash2 survey in France, and found to be in line with previous estimates[27,28]. In the results considered in the main paper, we did not consider further differences between the two strains (generation time, hospitalization or severity rate). Recent results confirm that infection with lineage B.1.1.7 was associated with an increased risk of hospitalization compared with other lineages (adjusted OR of 1.64 (95%CI, 1.32-2.04)). We then considered a 64% increase in hospitalization rates if infected with B.1.1.7 and refitted the model to hospitalization data to provide updated projections under this condition. Results are shown in section 4.

B.1.1.7 variant was initialized on January 7, 2021 (in w01) using the estimates of the first large-scale nationwide genomic surveillance survey (Flash1, see section 1 and main text). No other information was assumed, beyond the increased transmissibility advantage (and the increased hospitalization rate, for sensitivity). As such, no specific dynamics on the two strains is imposed *a priori*, and the trajectories predicted by the model are the result of the fit to hospitalization data (see next section).

2.5. Vaccination rollout campaign

Following estimated plans for rolling out the vaccination campaign, we simulated a rollout scenario based on the administration of 100,000 doses per day in France from w04, prioritized to the older age class. This was based on recent data, reporting an average daily rhythm of vaccination of 99,500 doses (including first and second doses) from w04 to w08[29]. We considered 75% vaccine efficacy against susceptibility[30], 65% vaccine efficacy against transmission[31], and a range between 40% and 80% for vaccine efficacy against symptoms given infection, computed from the estimated vaccine reduction of symptomatic disease[31,32], between 85%[30] and 95%[33,34]. Estimates were found to be similar when evaluated 14 days after the first dose or 1-2 weeks after the second dose[34], therefore we assumed efficacy to start 14 days after the first injection. We also considered for sensitivity: (i) a reduced efficacy against transmission (50%); (ii) no efficacy against susceptibility and 90% efficacy against symptoms.

Following the announcements by French authorities on March 4 aiming at administering 10 million first doses till mid-April[35], we considered an acceleration in the daily rhythm in w10 (starting March 8) with 200,000 doses per day (only first doses). For sensitivity, we considered a more optimistic rollout of 300,000 doses/day (only first doses; this latter estimate based on observations that 250,000 doses were administered on March 5 after the announcements[36]). These rollouts are compared to a stable rhythm of 100,000 doses (used for first and second injections).

3. Inference framework

Once the model is parameterized with the data described above, we infer the transmission rate per contact by fitting the model to daily hospital admission data through a maximum likelihood procedure in each pandemic phase. More precisely, prior to lockdown and in absence of intervention (period January-March 2020), we estimated $\{\beta, t_0\}$ where β is the transmission rate per contact and t_0 the date of the start of the simulation, seeded with 10 infectious individuals. Then, in each phase we estimated α_{phase}, i.e. the scaling factor of the transmission rate per contact specific to the pandemic phase under study (e.g. lockdown, exit from lockdown, summer, start of second wave, second lockdown, etc.). The transmission rate per contact in each phase is then defined as the transmission rate per contact in the pre-lockdown phase β multiplied by the scaling factor α_{phase}. A pandemic phase is defined by the interventions implemented (e.g. lockdown, curfew, and other restrictions) and activity of the population (school holidays, summer holidays, etc.).

We used simulations of the stochastic model to predict values for all quantities of interest (500 stochastic simulations each time). We fitted the model to the daily count of hospitalizations $H_{obs}(d)$ on day *d*. The likelihood function is of the form

$$L(Data|\Theta) = \prod_{t=t_1}^{t_n} Poiss\left(H_{obs}(t) \middle| H_{pred}(t)\right)$$

where Θ indicates the set of parameters to be estimated, $H_{obs}(t)$ is the observed number of hospital admissions on day t, $H_{pred}(t)$ is the number of hospital admissions predicted by the model on day t, $Poiss\left(\cdot \middle| H_{pred}(t)\right)$ is the probability mass function of a Poisson distribution with mean $H_{pred}(t)$, and $[t_1, t_n]$ is the time window considered for the fit. The effect of social distancing in January 2021 was estimated in the w02-w05 period, to account for the expected delay from the implementation of the measures (starting at the end of week 53, then progressively strengthened till w02) and hospitalizations.

Wald confidence intervals for the scaling factor α were computed by fitting a quadratic function on the loglikelihood values around the MLE, to estimate Fisher's information.

In prior work[1] we showed that the stochasticity of the model is the main source of uncertainty in the predictions.

Simulations for France and Nouvelle Aquitaine are initialized with 10 infected adults in the I_p compartment at the estimated time t_0 and progress over the entire 2020 to describe the full trajectory of the epidemic in France (and in each region under study) and build up the immunity in the population, prior to the arrival of B.1.1.7 variant. For Île-de-France, we seeded the model with 140 infected individuals at the corresponding estimated time t_0 to reduce the strong fluctuations associated with fitting the rapid increase and the high peak of hospitalizations observed in the first wave (the region was one of the areas strongly affected by the epidemic in early 2020). The model was validated against the estimates of three independent serological surveys conducted in France[1]. The second wave, under the effect of the second national lockdown, is shown in the Figures reporting the trajectory (main text and SI) to provide the epidemic context of COVID-19 pandemic in France, and a reference frame for the critical levels of hospitalizations that triggered the second lockdown.

4. Additional results and sensitivity analysis

4.1. Impact of B.1.1.7 transmissibility advantage

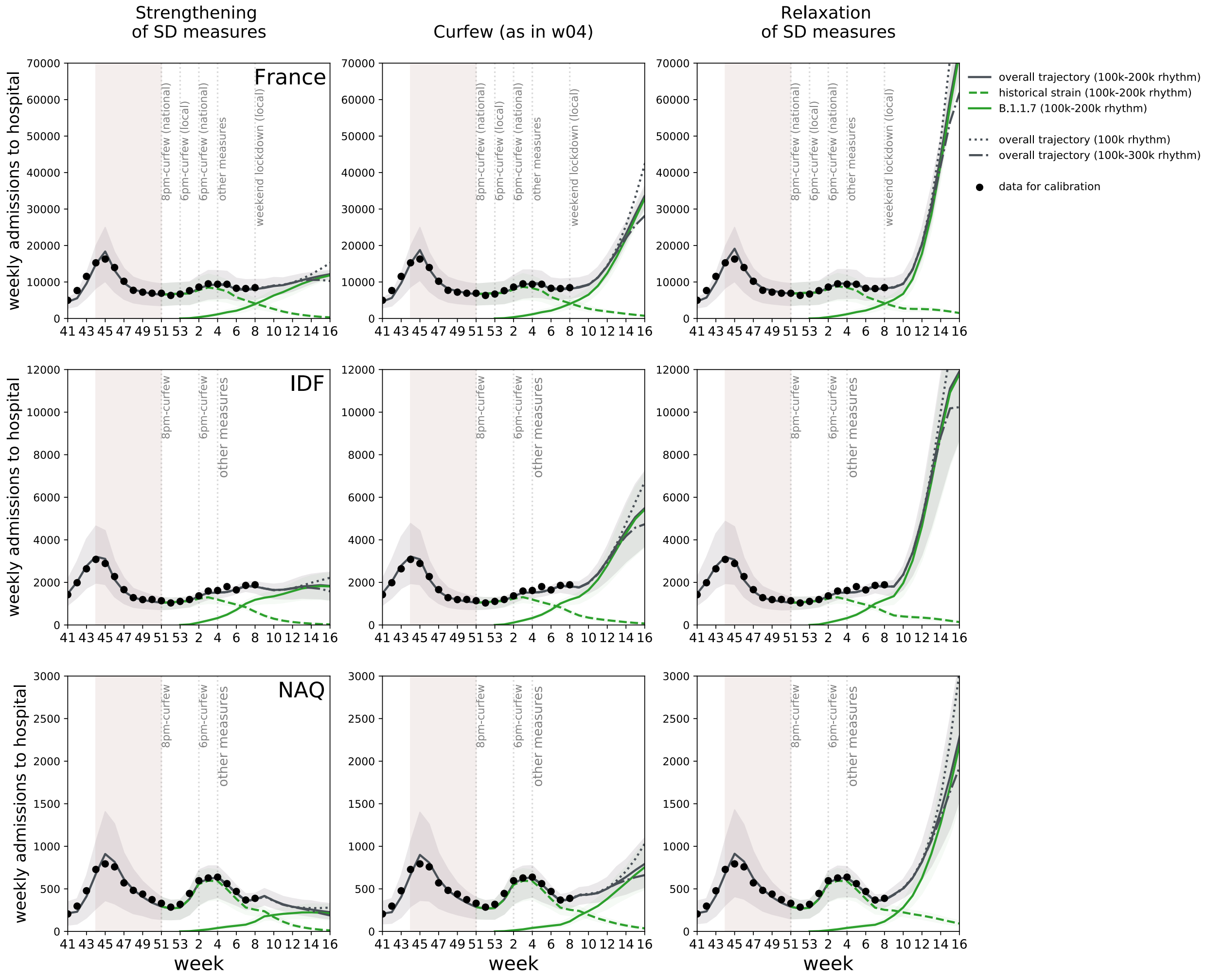

Figure S5. **Impact of 54% transmissibility increase on the projected weekly hospitalizations due to SARS-CoV-2 historical strain and B.1.1.7 variant.** From left to right, different scenarios considered after winter school holidays: strengthening of social distancing (SD) measures scenario, equivalent to the second lockdown; curfew scenario, estimated in w04 and assuming no additional changes; relaxation of SD measures scenario, compatible with the situation at the start of the year before increased restrictions. From top to bottom: France, Île-de-

France (IDF), Nouvelle Aquitaine (NAQ). The solid grey curve refers to the median overall trajectory, obtained under the accelerated vaccination rollout (100k-200k doses/day) and due to the concurrent circulation of the historical strain (dashed green curve) and B.1.1.7 variant (solid green curve), assuming 54% increase in transmissibility. A slower (100k, dotted curve) and optimistic (100k-300k, dot-dashed curve) vaccination rhythms are also shown (only median curves of the overall trajectories are shown, for the sake of visualization). The shaded area around the curves corresponds to the 95% probability range obtained from 500 stochastic simulations. Dots correspond to weekly hospital admission data. The model is fit to daily hospital admissions since the start of the epidemic, propagating uncertainty over time; the figure shows weekly data to simplify the visualization. The second wave is shown for reference, together with indications of the timing of social distancing measures; the shaded rectangle around the second wave corresponds to the second lockdown.

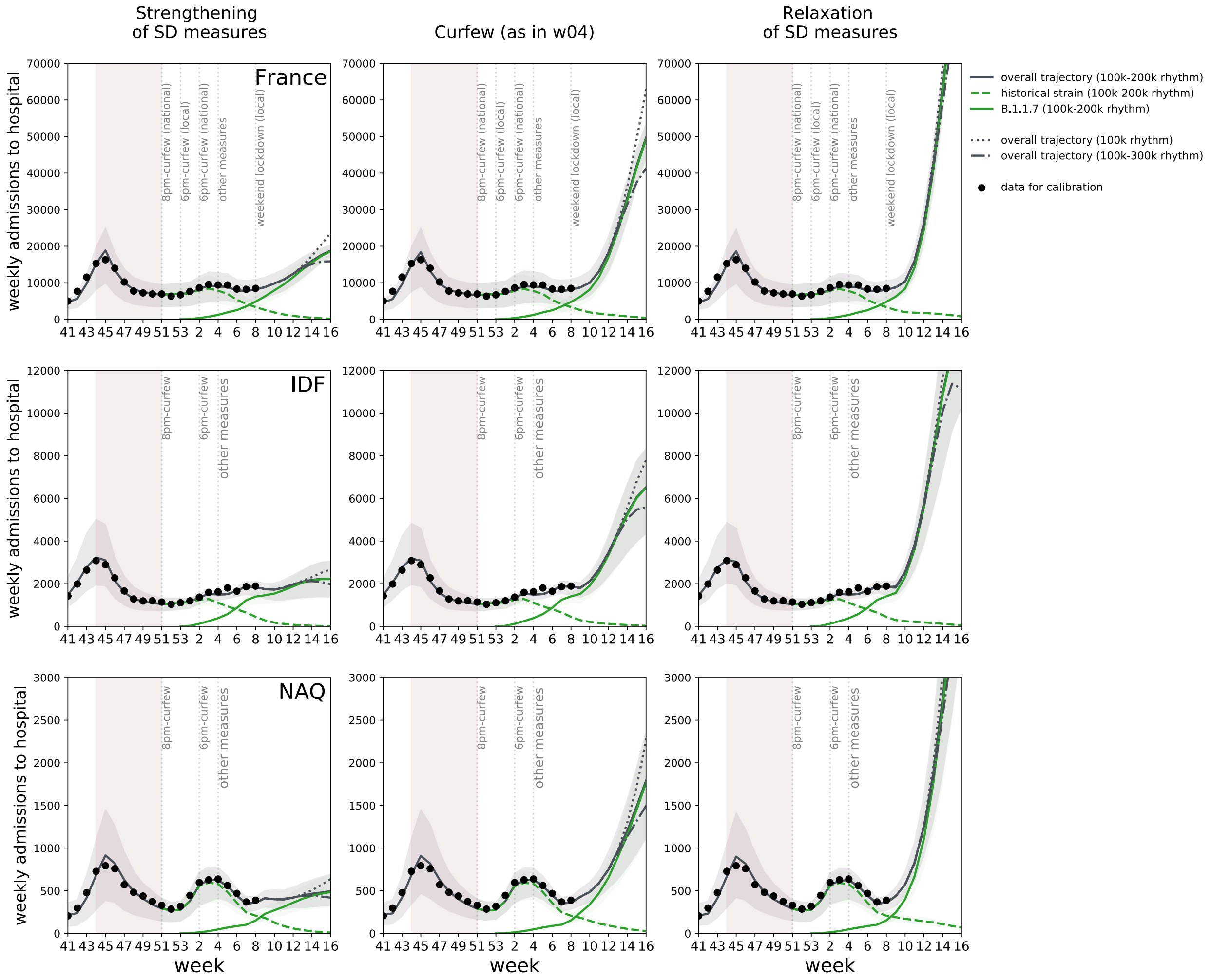

Figure S6. **Impact of 65% transmissibility increase on the projected weekly hospitalizations due to SARS-CoV-2 historical strain and B.1.1.7 variant.** From left to right, different scenarios considered after winter school holidays: strengthening of social distancing (SD) measures scenario, equivalent to the second lockdown; curfew scenario, estimated in w04 and assuming no additional changes; relaxation of SD measures scenario, compatible with the situation at the start of the year before increased restrictions. From top to bottom: France, Île-de-France (IDF), Nouvelle Aquitaine (NAQ). The solid grey curve refers to the median overall trajectory, obtained under the accelerated vaccination rollout (100k-200k doses/day) and due to the concurrent circulation of the historical strain (dashed green curve) and B.1.1.7 variant (solid green curve), assuming 65% increase in transmissibility. A slower (100k, dotted curve) and optimistic (100k-300k, dot-dashed curve) vaccination rhythms are also shown (only median curves of the overall trajectories are shown, for the sake of visualization). The shaded area around the curves corresponds to the 95% probability range obtained from 500 stochastic simulations. Dots correspond to weekly hospital admission data. The model is fit to daily hospital admissions since the start of the epidemic, propagating uncertainty over time; the figure shows weekly data to simplify the visualization. The second wave is shown for reference, together with indications of the timing of social distancing measures; the shaded rectangle around the second wave corresponds to the second lockdown.

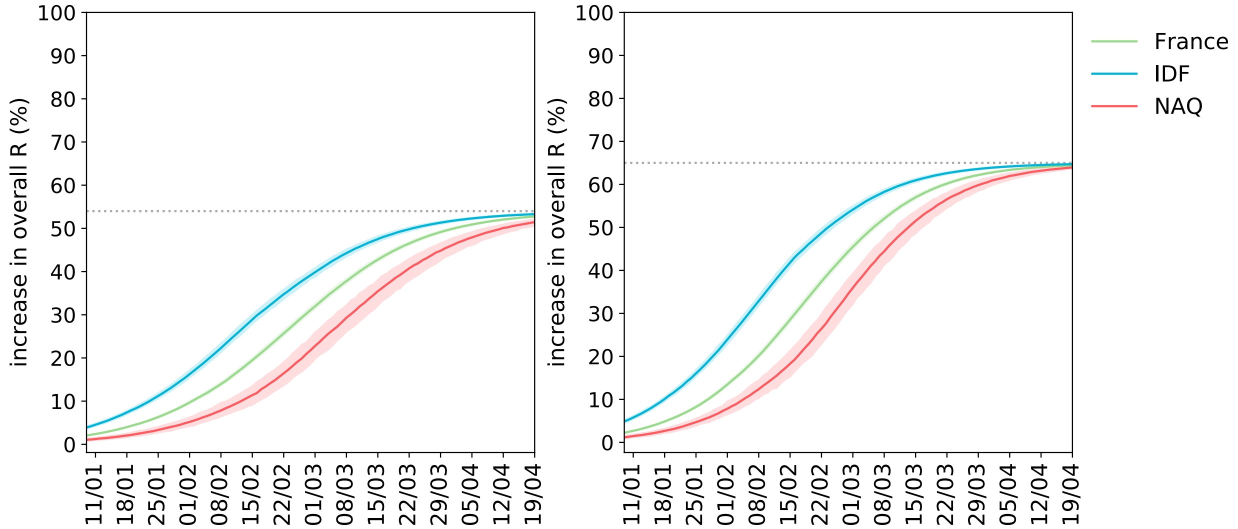

Figure S7. Percentage increase in the overall effective reproductive number at the population level due to the increased penetration of the variant. Results are shown for 54% transmissibility increase (left panel) and 65% transmissibility increase of the B.1.1.7 strain (right panel). Curves represent median values for France (green), Île-de-France (blue), Nouvelle Aquitaine (red); the shaded area around the curves corresponds to the 95% probability range obtained from 500 stochastic simulations.

4.2. Impact of strengthening / relaxation of social distancing measures on projected B.1.1.7 prevalence

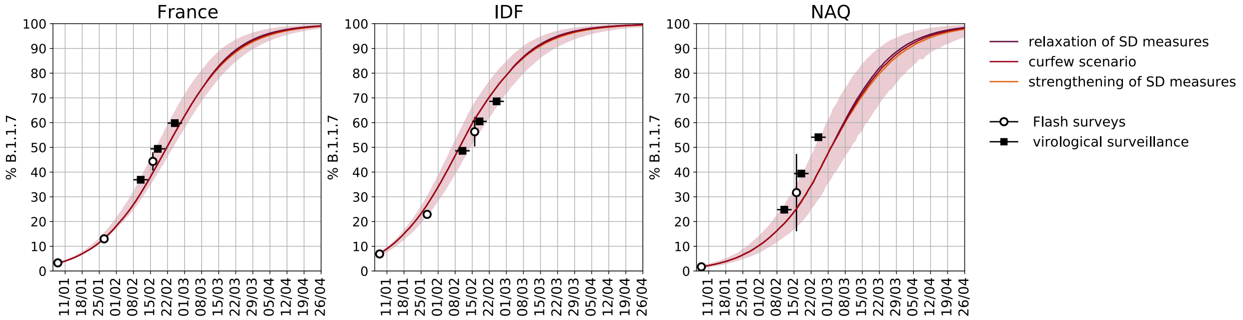

Figure S8. Impact of strengthening and relaxation of SD measures on the B.1.1.7 projected prevalence over time. Estimated percentage of B.1.1.7 cases over time, assuming 59% (95% CI: 54-65%) increase in transmissibility for the variant. From left to right: mainland France, Île-de-France region (IDF) and Nouvelle Aquitaine region (NAQ). Circles represent the estimates from the genomic surveillance of Flash surveys (Flash1 on January 7-8, Flash2 on January 27, Flash3 on February 16). Flash2 estimate is not reported for Nouvelle Aquitaine, as sequencing failed or was not performed on the majority of samples, preventing a reliable estimate. Squares represent results from weekly virological surveillance screening allowing the detection of the N501Y mutation specific to B.1.1.7 variant. 95% confidence intervals are estimated assuming a normal distribution. Flash 3 survey estimates have larger confidence intervals as sequencing was performed on a smaller sample of viruses. Horizontal error bars in weekly virological surveillance correspond to the week of reference. Curves represent median values; the shaded area around the curves corresponds to the 95% probability range obtained from 500 stochastic simulations. Color shade refers to the curfew scenario (intermediate color) and the scenarios with strengthening (lighter color) and relaxation (darker color) of social distancing measures. Results show that social distancing measures of this intensity do not have a significant impact on the prevalence of B.1.1.7 over time, as they concurrently act on both strains.

4.3. Impact of more restrictive social distancing measures on projected trends of hospital admissions

Here we present the results obtained under more restrictive measures, corresponding to a 20% reduction of the effective reproductive number estimated for the curfew in w04. Such reduction is estimated to be necessary to avoid a considerable increase in hospital admissions in France, and achieve a reduction in Île-de-France.

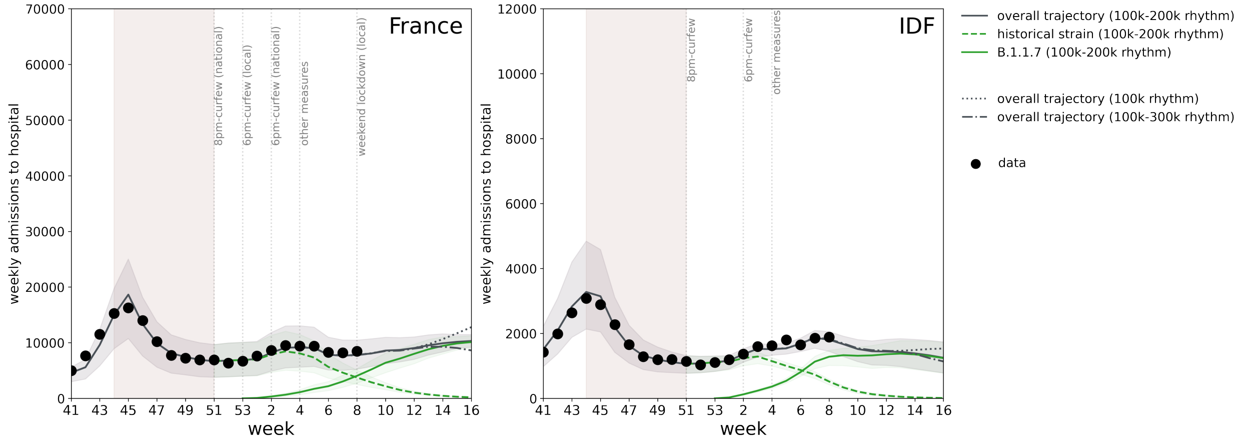

Figure S9. **Impact of 20% reduction of the effective reproductive number on the projected weekly hospitalizations due to SARS-CoV-2 historical strain and B.1.1.7 variant.** From left to right: mainland France, Île-de-France (IDF). Scenario considered after winter school holidays: strengthening of social distancing (SD) measures corresponding to a reduction of 20% of the effective reproduction number. The solid grey curve refers to the median overall trajectory, obtained under the accelerated vaccination rollout (100k-200k doses/day) and due to the concurrent circulation of the historical strain (dashed green curve) and B.1.1.7 variant (solid green curve), assuming 59% increase in transmissibility. A slower (100k, dotted curve) and optimistic (100k-300k, dot-dashed curve) vaccination rhythms are also shown (only median curves of the overall trajectories are shown, for the sake of visualization). The shaded area around the curves corresponds to the 95% probability range obtained from 500 stochastic simulations. Dots correspond to weekly hospital admission data. The model is fit to daily hospital admissions since the start of the epidemic, propagating uncertainty over time; the figure shows weekly data to simplify the visualization. The second wave is shown for reference, together with indications of the timing of social distancing measures; the shaded rectangle around the second wave corresponds to the second lockdown.

4.4. Sensitivity analysis on vaccine daily rhythm and efficacy

4.4.1. Impact of vaccination rhythms on the projected weekly hospitalizations

Figure S10 shows the effect of vaccination under different rhythms compared to a no vaccination scenario for France, and **Table S3** shows the expected week at which hospitalizations would reach the levels of the second wave, under the different social distancing scenarios and vaccination rhythms, for all territories.

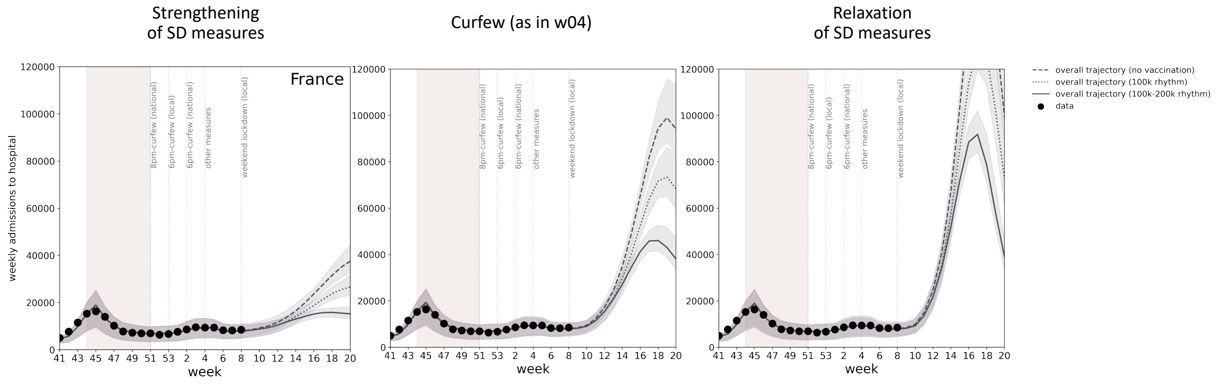

Figure S10. **Impact of vaccination rhythms on the projected weekly hospitalizations due to SARS-CoV-2 historical strain and B.1.1.7 variant.** Results are shown for mainland France. From left to right, different scenarios considered after winter school holidays: strengthening of social distancing (SD) measures scenario, equivalent to the second lockdown; curfew scenario, estimated in w04 and assuming no additional changes; relaxation of SD measures scenario, compatible with the situation at the start of the year before increased restrictions. Dots correspond to weekly hospital admission data. Curves refer to the expected trajectory with the accelerated vaccination rhythm (100k-200k doses/day, solid curve, as in the main paper), stable rhythm (100k doses/day, dotted), and no vaccination (dashed curve). The shaded area around the curves corresponds to the 95% probability range obtained from 500 stochastic simulations. Dots correspond to weekly hospital admission data. The model is fit to daily hospital admissions since the start of the epidemic, propagating uncertainty over time; the figure shows weekly data to simplify the visualization. The second wave is shown for reference, together with indications of the timing of social distancing measures; the shaded rectangle around the second wave corresponds to the second lockdown

Table S3. **Estimated week at which hospitalizations exceed the peak value of the second wave in France and in the two regions under study, for varying vaccination rollouts.** Projections after school holidays consider the curfew scenario (estimated before school holidays, assuming no additional interventions, central column), a scenario assuming a strengthening of social distancing (SD) measures (left column), and one assuming a relaxation of social distancing measures (right column). Results correspond to 59% increase of transmissibility of the variant. Results cannot integrate yet the effect of the weekend lockdowns in certain areas, nor Easter school holidays. Ranges refer to 95% probability ranges; "--" indicates that the peak level is not reached. Figures of daily administration of vaccine doses in the table refer to the national level. In each region daily rhythms are computed considering a population-weighted distribution for the senior age class.

	**Peak weekly hospitalizations of second wave**				
		**Vaccine doses per day from w10**	**Strengthening of SD measures**	**Curfew (as in w04)**	**Relaxation of SD measures**
**France**	16,000 weekly hospitalizations	100k rhythm	week 15 (14-16)	week 13 (12-13)	week 12 (11-12)
		200k rhythm	After week 15	week 13 (12-13)	week 12 (11-12)
		300k rhythm	--	week 13 (12-14)	week 12 (11-12)
**Île-de-France**	3,000 weekly hospitalizations	100k rhythm	--	week 12 (11-13)	week 11 (11-12)
		200k rhythm	--	week 12 (11-12)	week 11 (11-12)
		300k rhythm	--	week 12 (11-12)	week 11 (11-12)
**Nouvelle Aquitaine**	800 weekly hospitalizations	100k rhythm	After week 15	week 14 (13-16)	week 12 (11-13)
		200k rhythm	--	week 15 (13-19)	week 12 (11-13)
		300k rhythm	--	week 15 (13-19)	week 12 (11-13)

4.4.2. Impact of vaccine efficacy on the projected weekly hospitalizations

Figure S11 reports the sensitivity on vaccine efficacy.

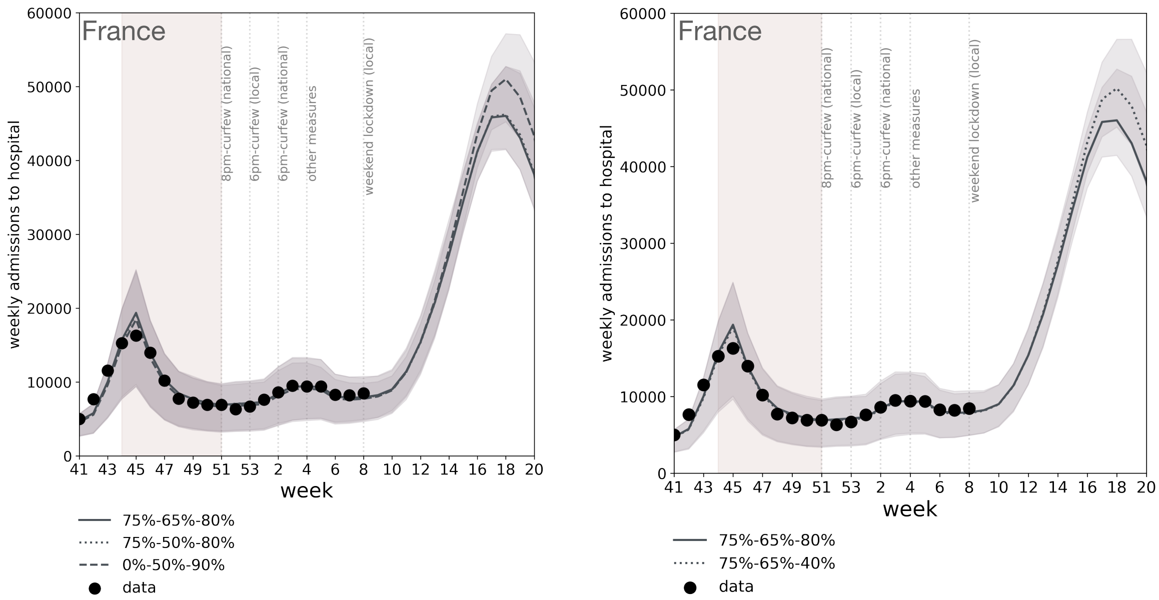

Figure S11. **Impact of vaccine efficacy on the projected weekly hospitalizations due to SARS-CoV-2 historical strain and B.1.1.7 variant.** Results are shown for mainland France. Scenario considered after winter school holidays: curfew scenario, estimated in w04 and assuming no additional changes. Curves refer to the median overall trajectory, obtained under the accelerated vaccination rollout (100k-200k doses/day) and due to the concurrent circulation of the historical strain and B.1.1.7 variant, assuming 59% increase in transmissibility. In both panels, solid line is obtained assuming a 75% vaccine efficacy against susceptibility, 65% vaccine efficacy against transmission and 80% vaccine efficacy against symptoms given infection, as in the main paper (indicated as 75%-65%-80% in the Figure legend). Left panel: dotted line is obtained assuming 75% vaccine efficacy against susceptibility, 50% vaccine efficacy against transmission and 80% vaccine efficacy against symptoms given infection (75%-50%-80%); dashed line is obtained assuming no efficacy against susceptibility, 50% vaccine efficacy against transmission, and 90% vaccine efficacy against symptoms given infection (0%-50%-90%). Right panel: dotted line is obtained assuming 75% vaccine efficacy against susceptibility, 65% vaccine efficacy against transmission and 40% vaccine efficacy against symptoms given infection (75%-65%-40%). Shaded area around the curves corresponds to the 95% probability range obtained from 500 stochastic simulations. Dots correspond to weekly hospital admission data. The model is fit to daily hospital admissions since the start of the epidemic, propagating uncertainty over time; the figure shows weekly data to simplify the visualization. The second wave is shown for reference, together with indications of the timing of social distancing measures; the shaded rectangle around the second wave corresponds to the second lockdown.

We note that our results may be an underestimation of the impact of vaccination on the epidemic trajectory as in our model priority is given to 65+ whereas vaccination is currently being rolled out first in the 75+ age class, characterized by a higher hospitalization rate. As our model does not break down further age classes above 65 years of age, vaccination cannot account for the higher advantage in targeting older age classes. On the other hand, we optimistically assume that vaccine efficacy is reached 2 weeks after the first injection, which was observed so far for mRNA-1273 vaccine[34].

4.5. Impact of increased hospitalization rates associated to B.1.1.7 infection

The increased hospitalization rate (+64%) after B.1.1.7 infection, recently estimated in Denmark[37], is expected to lead to a higher peak of hospital admissions starting at the end of April (**Figure S12**), if curfew measures only are in place. Hospital admission would be however largely higher than the levels of the first and second peak, even with the considered vaccination rhythms.

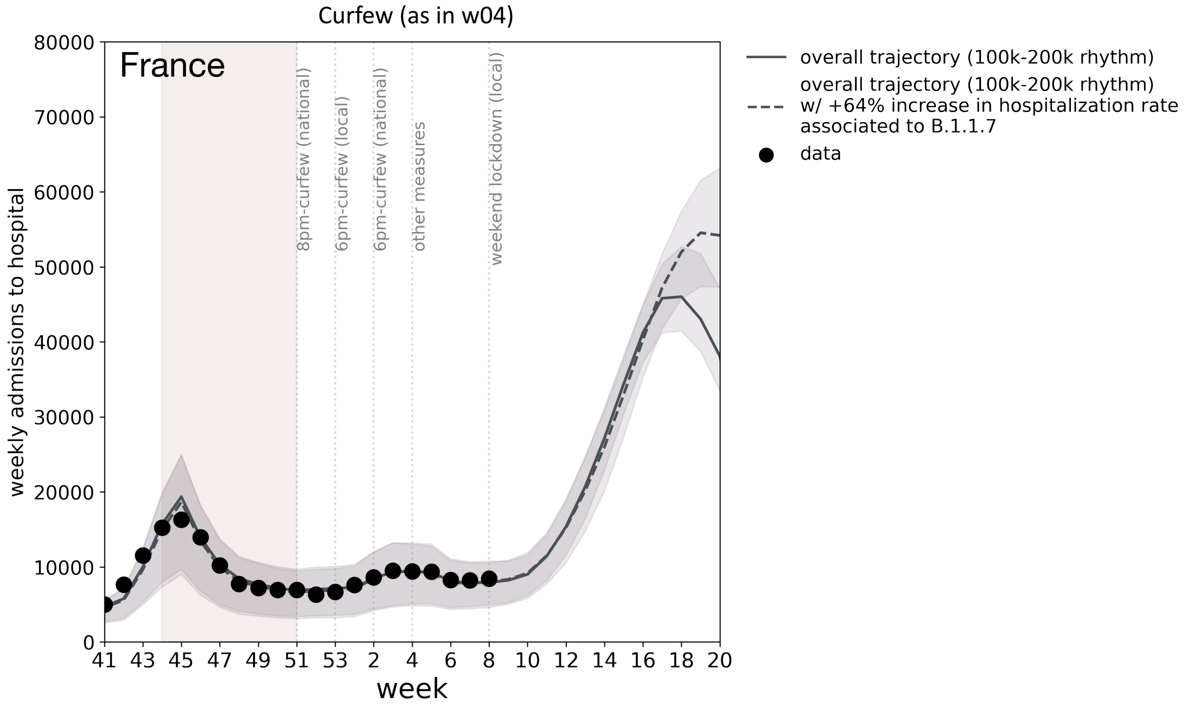

Figure S12. **Impact of an increased hospitalization rate after B.1.1.7 infection on the projected weekly hospitalizations due to SARS-CoV-2 historical strain and B.1.1.7 variant.** Results are shown for mainland France. Scenario considered after winter school holidays: curfew scenario, estimated in w04 and assuming no additional changes. Curves refer to the overall trajectory, due to the concurrent circulation of the historical strain and of the B.1.1.7 variant, assuming 59% increase in transmissibility and under the accelerated vaccination rollout (100k-200k doses/day). The solid grey curve refers to the median overall trajectory, obtained assuming the same hospitalization rates for infection due to the historical train and B.1.1.7. variant (as in the main paper). The dashed grey curve refers to the median overall trajectory, obtained assuming an increased hospitalization rate (+64%) associated to B.1.1.7 infection[37].The shaded area around the curves corresponds to the 95% probability range obtained from 500 stochastic simulations. Dots correspond to weekly hospital admission data. The model is fit to daily hospital admissions since the start of the epidemic, propagating uncertainty over time; the figure shows weekly data to simplify the visualization. The second wave is shown for reference, together with indications of the timing of social distancing measures; the shaded rectangle around the second wave corresponds to the second lockdown.

5. References

1. Pullano, G. *et al.* Underdetection of cases of COVID-19 in France threatens epidemic control. *Nature* **590**, 134–139 (2021).
2. Di Domenico, L., Pullano, G., Sabbatini, C. E., Boëlle, P.-Y. & Colizza, V. Impact of lockdown on COVID-19 epidemic in Île-de-France and possible exit strategies. *BMC Med.* **18**, 240 (2020).
3. Salje, H. *et al.* Estimating the burden of SARS-CoV-2 in France. *Science* **369**, 208–211 (2020).
4. Gaymard, A. *et al.* Early assessment of diffusion and possible expansion of SARS-CoV-2 Lineage 20I/501Y.V1 (B.1.1.7, variant of concern 202012/01) in France, January to March 2021. *Eurosurveillance* **26**, 2100133 (2021).
5. Gouvernement.fr. Plateforme COVID-19- tests. https://covid-19.sante.gouv.fr/tests.
6. Santé publique France. COVID-19 : point épidémiologique du 11 mars 2021. https://www.santepubliquefrance.fr/maladies-et-traumatismes/maladies-et-infections-respiratoires/infection-a-coronavirus/documents/bulletin-national/covid-19-point-epidemiologique-du-11-mars-2021.
7. Santé publique France. COVID-19 : point épidémiologique du 4 mars 2021. https://www.santepubliquefrance.fr/maladies-et-traumatismes/maladies-et-infections-respiratoires/infection-a-coronavirus/documents/bulletin-national/covid-19-point-epidemiologique-du-4-mars-2021.

8. Lavezzo, E. *et al.* Suppression of a SARS-CoV-2 outbreak in the Italian municipality of Vo'. *Nature* **584**, 425–429 (2020).
9. Riccardo, F. *et al.* Epidemiological characteristics of COVID-19 cases and estimates of the reproductive numbers 1 month into the epidemic, Italy, 28 January to 31 March 2020. *Eurosurveillance* **25**, 2000790 (2020).
10. Lapidus, N. *et al.* Do not neglect SARS-CoV-2 hospitalization and fatality risks in the middle-aged adult population. *Infect. Dis. Now* (2021) doi:10.1016/j.idnow.2020.12.007.
11. Li, R. *et al.* Substantial undocumented infection facilitates the rapid dissemination of novel coronavirus (SARS-CoV-2). *Science* **368**, 489 (2020).
12. Zhang, J. *et al.* Changes in contact patterns shape the dynamics of the COVID-19 outbreak in China. *Science* **368**, 1481–1486 (2020).
13. Davies, N. G. *et al.* Age-dependent effects in the transmission and control of COVID-19 epidemics. *Nat. Med.* **26**, 1205–1211 (2020).
14. Zimmermann, P. & Curtis, N. Coronavirus Infections in Children Including COVID-19: An Overview of the Epidemiology, Clinical Features, Diagnosis, Treatment and Prevention Options in Children. *Pediatr. Infect. Dis. J.* **39**, 355–368 (2020).
15. Jiehao, C. *et al.* A Case Series of children with 2019 novel coronavirus infection: clinical and epidemiological features. *Clin. Infect. Dis.* **71**, 1547–1551 (2020).
16. Fontanet, A. *et al.* SARS-CoV-2 infection in primary schools in northern France: A retrospective cohort study in an area of high transmission. *medRxiv* http://medrxiv.org/lookup/doi/10.1101/2020.06.25.20140178 (2020).
17. Fontanet, A. *et al.* Cluster of COVID-19 in northern France: A retrospective closed cohort study. *medRxiv* https://www.medrxiv.org/content/10.1101/2020.04.18.20071134v1 (2020).
18. Van Kerckhove, K., Hens, N., Edmunds, W. J. & Eames, K. T. D. The impact of illness on social networks: implications for transmission and control of influenza. *Am. J. Epidemiol.* **178**, 1655–1662 (2013).
19. Di Domenico, L., Pullano, G., Sabbatini, C. E., Boëlle, P.-Y. & Colizza, V. Modelling safe protocols for reopening schools during the COVID-19 pandemic in France. *Nat. Commun.* (2021) doi:10.1101/2020.05.08.20095521.
20. Lauer, S. A. *et al.* The Incubation Period of Coronavirus Disease 2019 (COVID-19) From Publicly Reported Confirmed Cases: Estimation and Application. *Ann. Intern. Med.* **172**, 577–582 (2020).
21. Ferretti, L. *et al.* Quantifying SARS-CoV-2 transmission suggests epidemic control with digital contact tracing. *Science* **368**, eabb6936 (2020).
22. Cereda, D. *et al.* The early phase of the COVID-19 outbreak in Lombardy, Italy. *arXiv:2003.09320 [q-bio]* http://arxiv.org/abs/2003.09320 (2020).
23. Svensson, A. A note on generation times in epidemic models. *Math. Biosci.* **208**, 300–311 (2007).
24. Ministère de l'Education Nationale de la Jeunesse et des Sports. Déconfinement phase 2 : point de situation au 28 mai. *Ministère de l'Education Nationale et de la Jeunesse* https://www.education.gouv.fr/deconfinement-phase-2-point-de-situation-au-28-mai-303813.
25. Google.com. COVID-19 Community Mobility Report. *COVID-19 Community Mobility Report* https://www.google.com/covid19/mobility?hl=fr.
26. Santé publique France. Covid-19 : une enquête pour suivre l'évolution des comportements et de la santé mentale pendant l'épidémie. https://www.santepubliquefrance.fr/etudes-et-enquetes/covid-19-une-enquete-pour-suivre-l-evolution-des-comportements-et-de-la-sante-mentale-pendant-l-epidemie.
27. Davies, N. G. *et al.* Estimated transmissibility and impact of SARS-CoV-2 lineage B.1.1.7 in England. *Science* **372**, (2021).
28. Volz, E. *et al.* Assessing transmissibility of SARS-CoV-2 lineage B.1.1.7 in England. *Nature* 1–17 (2021). doi:10.1038/s41586-021-03470-x.
29. data.gouv.fr. Données relatives aux personnes vaccinées contre la Covid-19 (VAC-SI) - data.gouv.fr. /fr/datasets/donnees-relatives-aux-personnes-vaccinees-contre-la-covid-19-1/.
30. Amit, S., Regev-Yochay, G., Afek, A., Kreiss, Y. & Leshem, E. Early rate reductions of SARS-CoV-2 infection and COVID-19 in BNT162b2 vaccine recipients. *The Lancet* **397**, 875–877 (2021).
31. Lipsitch, M. & Kahn, R. Interpreting vaccine efficacy trial results for infection and transmission. *medRxiv* 2021.02.25.21252415 (2021) doi:10.1101/2021.02.25.21252415.
32. Halloran, M. E., Struchiner, C. J. & Longini, I. M., Jr. Study Designs for Evaluating Different Efficacy and

Effectiveness Aspects of Vaccines. *Am. J. Epidemiol.* **146**, 789–803 (1997).
33. Polack, F. P. *et al.* Safety and Efficacy of the BNT162b2 mRNA Covid-19 Vaccine. *N. Engl. J. Med.* **383**, 2603–2615 (2020).
34. Baden, L. R. *et al.* Efficacy and Safety of the mRNA-1273 SARS-CoV-2 Vaccine. *N. Engl. J. Med.* **384**, 403–416 (2021).
35. Gouvernement.fr. Discours de Jean Castex : conférence de presse sur les mesures contre la Covid-19. *Gouvernement.fr* https://www.gouvernement.fr/partage/12148-discours-de-jean-castex-conference-de-presse-sur-les-mesures-contre-la-covid-19.
36. BFMTV.com. Vaccin: près de 250.000 injections en une journée, un record depuis le début de la campagne. *BFMTV* https://www.bfmtv.com/sante/vaccin-pres-de-250-000-injections-en-une-journee-un-record-depuis-le-debut-de-la-campagne_AN-202103050406.html.
37. Bager, P. *et al.* Increased Risk of Hospitalisation Associated with Infection with SARS-CoV-2 Lineage B.1.1.7 in Denmark. https://papers.ssrn.com/abstract=3792894 (2021) doi:10.2139/ssrn.3792894.
